# Development of a sleep quality questionnaire to assess sleep in the ICU: a polysomnography study

**DOI:** 10.1186/cc12621

**Published:** 2013-06-19

**Authors:** LJ Storti, DM Servantes, MA Borges, FU Maroja, PR Burke, LR Bittencourt, ACC Carvalho, S Tufik, AAV de Paola, FD Cintra

**Affiliations:** 1 Escola Paulista de Medicina - Universidade Federal de São Paulo, Vila Clementino, São Paulo, SP, Brazil

## Introduction

Sleep is an important issue for the maintenance of cardiovascular homeostasis through heart rate and blood pressure modulation. Patients admitted in a coronary care unit (CCU) may exhibit a peculiar sleep pattern that is not fully understood. A feasible and cost-effective tool to analyze sleep in this scenario could bring important information for clinicians. The aim of this study was to evaluate sleep with a questionnaire developed specifically for the CCU and to establish correlations with polysomnography.

## Methods

Consecutive acute coronary syndrome patients admitted to a CCU between March 2011 and October 2012 were selected. The exclusion criteria were: hemodynamic instability, sedation, receiving vasoactive drugs, or under ventilation support. Patients were submitted to polysomnography in the first 36 hours after admission. A specific 18-question questionnaire (Storti questionnaire) divided into diurnal and nocturnal sleep was developed according to experts' skills. The Pittsburgh and the Storti questionnaires were applied immediately before the CCU discharge. Cronbach's alpha test was used for internal questionnaire validation. Spearman and Kruskal-Wallis test were used to analyze the correlation between polysomnography variables and questionnaire.

## Results

Ninety-nine patients (68% male; mean age 56 ± 10 years) were included. The mean BMI was 27 ± 5 kg/m^2^. Arterial hypertension was observed in 52% of the sample; 17% had diabetes, 39% were smokers. The patients present a total sleep time of 265 ± 81 minutes during the polysomnography, sleep efficiency was 62 ± 18%, REM sleep was 10 ± 7%, and apnea/hypopnea index and arousal index was 15 ± 23 and 24 ± 15, respectively. Cronbach's test was 0.69 (range: 0.63 to 0.69 when one of the questions was removed). The Storti questionnaire also showed correlations with polysomnography (*r *= 0.52; *P <*0.001), better than the Pittsburgh questionnaire (*r *= -0.25; *P *= 0.02). When sleep profile was divided according to the questionnaire score into three categories - poor, regular and good - poor and regular sleep was observed in 64 (65%) patients and good sleep in 35 (35%) patients. Moreover, patients classified as good sleepers had a sleep efficiency of 72 ± 9%, better than those with a regular or poor sleep (60 ± 16% and 53 ± 20%, respectively; *P <*0.01).

## Conclusion

The Storti questionnaire had a good correlation with sleep efficiency assessed by polysomnography. The majority of patients in CCU had a poor or regular sleep.

